# The Human–Horse Relationship: Identifying the Antecedents of Horse Owner Attitudes towards Horse Husbandry and Management Behaviour

**DOI:** 10.3390/ani11020278

**Published:** 2021-01-22

**Authors:** Lauren M. Hemsworth, Ellen C. Jongman, Grahame J. Coleman

**Affiliations:** Animal Welfare Science Centre, Faculty of Veterinary and Agricultural Sciences, University of Melbourne, Parkville, VIC 3010, Australia; ejongman@unimelb.edu.au (E.C.J.); grahame.coleman@unimelb.edu.au (G.J.C.)

**Keywords:** recreational horse ownership, human–animal relationship, demographics, knowledge, experience, attitudes to husbandry and management practices, behaviour, horse welfare

## Abstract

**Simple Summary:**

The welfare of recreational horses is an important issue worldwide. Since horse owner characteristics may be an important determinant of horse welfare, the relationships between horse owner background factors and horse owner attitudes towards horse husbandry and management behaviour were examined in this study. Owner knowledge and experience rather than demographics were associated with an appreciation of parasite control, hoof care, and dental care. Therefore, an educational strategy intended to improve the horse owner’s appreciation of the welfare implications of their behaviour may be important in safeguarding recreational horse welfare, but further research is warranted.

**Abstract:**

The welfare of recreational horses is an important issue. Horse owner attitudes towards horse ownership are likely to influence owner behaviour in terms of horse husbandry and management practices and human–horse interactions, which in turn are likely to affect the welfare of the horse. Based on Ajzen’s Theory of Planned Behaviour, this paper examines the relationships between horse owner attributes, specifically background factors (demographics, knowledge, and experience) and attitudes (beliefs) towards horse husbandry and management behaviour. Data were collected via a random telephone survey and during on-site inspections with Victorian horse owners and their horses (*n* = 57), using an attitude questionnaire. Relationships were found between horse owner background factors and horse owner attitudes towards horse husbandry and management behaviour. Generally, belief variables correlated significantly with background factors that were primarily related to knowledge and experience. Further, beliefs concerning three key husbandry practices (parasite control, hoof care, and dental care) all appear to be predicted to some degree by background factors associated with knowledge and experience. Therefore, a practical recommendation may be the implementation of education and training programs aimed at improving horse owner knowledge and experience regarding effective horse husbandry and management to promote horse welfare. Clearly, further research is warranted.

## 1. Introduction

The welfare of recreational horses is an important issue both in Australia and worldwide. The Australian Horse Industry Council (AHIC) defines a recreational horse as any horse not currently involved within the horse racing industry (both Thoroughbred and Standardbred racing). The recreational horse industry can be further defined according to its competitive and non-competitive nature; the competitive form involves disciplines including dressage, show jumping, eventing, showing, endurance riding and polo, and the non-competitive form includes leisure riding and companionship. A substantial proportion of the welfare problems observed in both local and international recreational horse populations are due to horse owner mismanagement, as a result of ignorance rather than intentional abuse [1,2,3,4,5,6]. Inappropriate husbandry and management include inadequate provision of feed, provision of unsuitable feed, inadequate hoof care, inadequate parasite control, failure to seek appropriate veterinary attention for illness or injury, inadequate dental care, inappropriate housing, and insufficient exercise [1,2,4,5,6,7,8,9,10,11,12,13]. The literature, whilst limited, identifies possible relationships between horse owner attributes and horse welfare outcomes [1,4,14,15,16].

The sequential relationship between human attitudes and behaviour described by Ajzen’s Theory of Planned Behaviour (TPB) [17] has been shown in the pork, poultry, veal, and dairy industries (see reviews [18,19,20]). In this research, stockperson attitudes were correlated with stockperson behaviour, which in turn were correlated with animal productivity and welfare. In addition to the role of attitudes, the manner in which a person behaves when managing their animals may be influenced by a number of other factors including social pressures, environment, motivation, knowledge and skills, and the satisfaction gained from animal interaction [19]. Hemsworth et al. [5] suggest that whilst there are likely to be some differences between the specific attitudes and behaviour of livestock stockpeople and horse owners, a similar human–horse relationship may exist, but this warrants investigation.

The research investigating the human–animal relationship (HAR) in livestock has focused primarily on the influence that human–animal interactions associated with daily handling have on the welfare and productivity of farm animals. However, research suggests that while handling of horses is most likely important in determining the horse’s response to the horse owner and other humans [13,21,22,23], the husbandry and management behaviour of horse owners may also be a significant determinant of the welfare of their horses. It should also be recognised that the HAR research in livestock indicates relationships between human attitudes and behaviour and other job-related characteristics including job satisfaction, work motivation, and motivation to learn, all of which may affect the husbandry and management behaviour of stockpeople [24]. There are undoubtedly differences between the HARs both within the livestock industries as well as between the livestock and recreational horse industries with regard to the nature and duration of the human interaction or behaviour and the degree of knowledge regarding appropriate husbandry and management practices [5]. However, both relationships are still likely to be based on the same underlying principles, that is, the quality and frequency of interactions between the two individuals, as well as the context in which they occur, will determine the quality of the HAR which, in turn, has implications for both human behaviour and work-related characteristics [5,19].

Horse owners’ attitudes towards horse ownership are likely to influence their behaviour in terms of the implementation of horse husbandry and management practices and human–horse interactions. Subsequently, these horse owner behaviours are likely to affect the welfare of the horse. The antecedents of horse owner attitudes may include a range of background factors including demographics, horse ownership experience, knowledge, and commitment. Thus, based on the research from the livestock industries and the limited research available regarding the role of human–horse relationships (for example [15,23]), a number of horse owner attributes are potentially associated with horse welfare. The human component of this relationship involves both behavioural and psychological aspects and as such, the horse owner attributes to be examined should include background factors, attitudinal variables, and behavioural variables. With regard to horse-based variables, those concerning horse health and welfare are obvious inclusions for investigation. A recent study by Rowland et al. [23] investigated some belief and welfare variables in horses owned by Irish travellers. However, the focus was on knowledge and use of body condition scoring, and a limited number of questions about exercise, horse-owner bonding, horse capacity for feelings, and some of the problems with horse ownership. The research did not assess the kinds of attitudes proposed by Hemsworth et al. [5], i.e., horse owner salient beliefs about horses, horse husbandry, and management practices and human–horse interactions, or the relationships between human attitudes and behaviour and furthermore, may not be generalisable to recreational horse owners.

**Figure 1:**
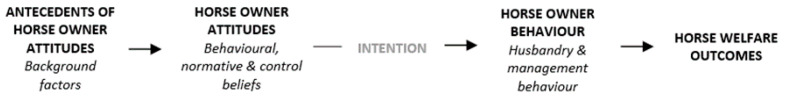
The hypothesised sequential relationships between recreational horse owner attributes in the human–horse relationship, and the ensuing relationship with recreational horse welfare [5,25].

Adapted from the TPB [17], a hypothesised HAR framework for recreational horse ownership was proposed by Hemsworth et al. [5]. The proposed human–horse relationship model describes the antecedents of horse owner husbandry and management behaviour and the ensuing relationship with horse welfare [5]. The current paper is one of two papers examining the sequential relationship between antecedents of horse owner attitudes, horse owner attitudes, horse owner behavior, and horse welfare. This first paper addresses the first aspect of the proposed human–horse relationship model (Figure 1), examining relationships between horse owner background factors including demographics, knowledge and experience, and horse owner attitudes (beliefs) towards horse husbandry and management behaviour. As Luna and Tadich [16] stated “To date, there is still a paucity of research dedicated to the identification and assessment of the human psychological attributes that affect the owner–equine interaction, and how these could affect the welfare of working equids”.

## 2. Materials and Methods

### 2.1. Study Design

This study was part of a large-scale investigation into the welfare of recreational horses in Victoria, Australia [25]. Data were collected during on-site inspections with Victorian horse owners and their horses between May (late autumn) and December (early summer), using the on-site inspection protocol which consisted of an attitude questionnaire followed by a horse and horse husbandry and management inspection. The on-site inspection collected attitudinal and behavioural data from horse owners and horse-based health and welfare outcomes from their horses. Up to two horses per owner were studied (H1 and H2). On average, the on-site inspection took approximately 120 min to complete. The sample consisted of 57 horse owners and 98 horses (H1 *n* = 57, H2 *n* = 41). The selection criteria required participants to be Victorian horse owners over 12 years of age who provided their horses with their daily primary care.

This project was approved by both Monash University’s human (MUHREC; CF07/0303–2007/0103) and animal (MARP AEC; SPPPM/2008/01-S1) ethics committees.

### 2.2. Participant Recruitment

Participants were initially recruited via a telephone survey investigating recreational horse ownership in Victoria, Australia [25]. A questionnaire was delivered to horse owners (*n* = 200) by I-View, a specialised market and social research data collection agency, using a combination of random digit dialling (RDD) telephone recruitment (*n* = 172) and probability internet panel recruitment (*n* = 28). The aim was to determine prevalence of recreational horse ownership in Victoria, to collect data on horse owner attributes and to then seek respondents’ participation in follow-up research to directly observe horse owner management behaviours and horse welfare, and collect data on horse owner attitudes (salient beliefs) towards horse husbandry and management behaviour and human–horse interactions.

The random telephone questionnaire collected data concerning horse owner background factors (demographics, knowledge, and experience), horse owner management behaviour, horse owner general attitudes, human–horse interactions, and horse demographic details. The questionnaire comprised seven sections and consisted of 123 questions (Table 1).

The random telephone questionnaire took approximately 40 min to complete. At the conclusion of the questionnaire, respondents were asked about their interest in participating in subsequent research. If the respondent was willing to participate in an on-site inspection, an appropriate time was determined and location details were recorded.

### 2.3. Data Collection: Horse Owner Attributes

The on-site inspections were completed by 57 horse owners (*n* = 57). The horse owner variables collected during both the random telephone survey (primarily the horse owner background factors) and the on-site inspection were grouped according to the type of variable. The horse owner variables that were examined with regard to their relationship to horse welfare included:

– horse owner background factors (demographic factors, knowledge, and experience),
– horse owner attitudes (behavioural, normative, and control beliefs) towards behaviour, and
– horse owner husbandry and management behaviour.

On-site inspection data were collected by the primary researcher (L.M.H), who received training for both the delivery of the attitude questionnaire and the assessment of horse welfare measures.

#### 2.3.1. Horse Owner Background Factors

Horse owner background factors largely consisted of demographic, knowledge and experience variables that were collected during the random telephone survey.

#### 2.3.2. Horse Owner Attitudes

The attitude questionnaire (*n* = 57) was delivered during the on-site inspection and measured multiple aspects of horse owner attitudes (beliefs) towards horses, related practices, and human–horse interactions, using the three types of attitude statements based on behavioural beliefs, normative beliefs, and control beliefs derived from the TPB [17].

- Behavioural beliefs (attitude towards a behaviour): measuring an individual’s positive or negative assessment of performing particular equine husbandry or management practice.
- Normative beliefs (subjective norms): measuring an individual’s perception of society’s approval or disapproval regarding the performance of a particular equine husbandry or management practice.
- Control belief (perceived behavioural control): measures an individual’s perceived ease or difficulty regarding the performance of a certain equine husbandry or management practice.

Using a 5-point Likert scale, horse owners were asked to indicate their level of agreement with the attitude statement or the level of importance they placed on the attitude statement. The use of multiple statements concerning a particular management practice allowed consistent beliefs relating to that topic to be identified and therefore the attitude toward the topic was able to be inferred [19].

The attitude questionnaire was developed following an extensive review of the literature and the horse husbandry and management practices investigated were based on the best practice guidelines outlined in the Codes of Practice pertaining to the welfare of horses [26].

##### The Development of Attitude Subscales

The attitude questionnaire contained 146 attitude items. It was not possible to reduce these items using principal component analysis (PCA) because the number of items in the questionnaire was greater than the sample size [27]. Therefore, to reduce the questionnaire data to a more appropriate size for data analysis, the items were grouped into attitude subscales with similar content. The items within each subscale were summed to create a single score for each subscale. Each attitude statement within a given attitude subscale was designed to measure a similar topic, however, to account for the different scale types (i.e., importance versus frequency versus agreement), all item scores were converted to Z-scores before summing. From the 146 items measured in the attitude questionnaire, 113 items were reduced into 12 attitude subscales (Table 2, Table 3, Table 4 and Table 5). All of the variables in a subscale were significantly correlated at a significance level of 0.01 with the subscale total, and all attitude subscales had a Cronbach’s alpha coefficient greater than 0.7 [27,28]. Thirty-three items were not grouped into an attitude subscale due to poor item-total correlations, and as a result, were treated as individual belief variables during subsequent data analysis.

The labelling of each attitude subscale was based on semantic content. The attitude subscales and the Cronbach’s alpha coefficients are presented in Table 2, Table 3, Table 4 and Table 5.

### 2.4. Statistical Analysis

Data were analysed using the statistical program IBM SPSS Statistics for Windows (Version 25, IBM Corp, Armonk, NY, USA). Data screening was performed on the complete data file to ensure the integrity and accuracy of the data prior to analysis.

#### 2.4.1. Relationships between Horse Owner Attributes: Horse Owner Background Factors and Horse Owner Belief Variables

Previous literature indicates that horse owner background factors and attitudes towards behaviour are associated with horse welfare outcomes. However, it is likely that most horse owner attributes do not share a direct relationship with horse welfare outcomes. Rather, horse owner attributes appear likely to form part of a sequential relationship as hypothesised in Figure 1. In accordance with the hypothesised model, a large number of Pearson product-moment correlation analyses were computed between horse owner background factors and horse owner belief variables, as the objective was not to examine specific relationships between individual variables but rather patterns between key horse owner attributes.

#### 2.4.2. Predicting Horse Owner Beliefs about Husbandry and Management Behaviour from Horse Owner Background Factors

Ideally, the interrelationships between the variables in the hypothesised human–horse relationship model (Figure 1) would be investigated using structural equation modelling. However, the sample size did not permit this, and as a result, the interrelationships were examined using a series of stepwise linear regressions relevant to each stage of the model. The variables identified in the first section of the paper as being significantly correlated (with a significance level of *p* < 0.05) with the relevant dependent variable were used as independent variables in these regression analyses. This was to permit the unique contributions of these variables to the variance in the relevant dependent variable to be determined; that is, the relative contribution of the background factors to the variability in attitudinal beliefs (belief variables) towards the performance of three key husbandry behaviours: parasite control behaviour, hoof care behaviour, and dental care behaviour.

## 3. Results

### 3.1. Demographics of Sample

The horse owner background factors (demographic, knowledge and experience variables) are reported in Table 6. In addition, approximately 50% of participants had owned horses for more than 10 yr and less than 10% of participants had owned horses for less than six yr. Approximately 25% of horse owners owned only one horse, however, on average participants owned two horses. Approximately 20% of participants did not daily interact with their horse, and participants most commonly spent less than 30 min per d interacting with their horses.

The demographic characteristics of the horses studied and the descriptive data of the horse husbandry and management practices implemented by horse owners are reported in Table 7 and Table 8, respectively. Approximately 30% of horses had some form of disease, injury or illness as ascertained by the primary researcher (L.M.H). The primary researcher determined that none of the observed horse welfare concerns warranted reporting to the relevant welfare authority, i.e., all horses had a BCS above two and were receiving treatment for any disease, injury or illness.

### 3.2. Relationships between Horse Owner Attributes: Horse Owner Background Factors and Horse Owner Belief Variables

The significant Pearson-product moment correlations between background factors and attitude subscales, individual behavioural belief variables, individual normative belief variables, and individual control belief variables are reported in Table 9 (demographic-based background factors) and Table 10 (knowledge and experience-based background factors). These variables, that significantly correlated with the relevant dependent variable, were subsequently used as independent variables in the regression analyses in Section 3.3.

The attitude subscales horse husbandry and management–housing (BB), horse husbandry and management–health and welfare (NB), horse husbandry and management–housing (CB), human–horse interaction (NB), horse husbandry and management–diet (CB), and human–horse interaction (CB) were not significantly associated with any of the horse owner background factors.

### 3.3. Predicting Horse Owner Beliefs about Husbandry and Management Behaviour from Horse Owner Background Factors

The relative contribution of the background factors to attitudinal beliefs towards the three key husbandry behaviours was investigated and is reported in Table 11.

#### 3.3.1. Horse Owner Background Factors and Horse Owner Beliefs towards Parasite Control Behaviour

The relationships between background factors and beliefs towards parasite control behaviour are reported in Table 11. *Age* and *Riding instruction frequency* were predictive of behavioural beliefs about parasite control behaviour, accounting for 31% of the variance in the belief. *Age* (β = −0.48, *p* = 0.01) accounted for 20% of the variance and *Riding instruction frequency* (β = −0.43, *p* = 0.02) accounted for an additional 11%. The negative beta values from the behavioural belief variable *A28^(^*^-ve RC)^ resulted from a re-coded negative attitude item. These findings indicate that favourable behavioural beliefs towards appropriate parasite control are associated with a young age and frequent riding instruction.

*Region type* and *Age* were predictive of normative beliefs about parasite control behaviour, accounting for 13% of the variance in the belief. *Region type* (β = 0.33, *p* = 0.01) accounted for 9% of the variance and *Age* (β = 0.26, *p* = 0.04) accounted for an additional 4%. These results imply that favourable normative beliefs towards parasite control are associated with an urban region of primary residence and a young age.

*Age* and *Region type* were predictive of control beliefs regarding parasite control behaviour, accounting for 40% of the variance in the belief. *Age* (β = 0.59, *p* < 0.01) accounted for 32% of the variance and *Region type* (β = 0.33, *p* = 0.02) accounted for a further 9%. These findings suggest that favourable control beliefs regarding parasite control behaviour is associated with a young age and an urban primary residence in horse owners.

#### 3.3.2. Horse Owner Background Factors and Horse Owner Beliefs towards Hoof Care Behaviour

The relationships between background factors and horse owner beliefs towards hoof care behaviour are presented in Table 11. *Region type* and *Age* were predictive of behavioural beliefs about hoof care behaviour, accounting for 23% of the variance in the belief. *Region type* (β = 0.37, *p* < 0.01) accounted for 12% of the variance and *Age* (β = 0.35, *p* < 0.01) accounted for 11% of the variance. These findings indicate that favourable behavioural beliefs towards hoof care behaviour are associated with an urban region of primary residence and a young age.

*Age* (β = 0.42, *p* < 0.01) was predictive of normative beliefs about hoof care behaviour, accounting for 15% of the variance in the belief. This finding implies that a favourable normative belief about hoof care behaviour is associated with a young age.

*Age* and *Horse club and society membership* were predictive of control beliefs about hoof care behaviour, accounting for 16% of the variance in the belief. *Age* (β = 0.3, *p* < 0.01) accounted for 9% of the variance and *Horse club and society membership* (β = 0.29, *p* < 0.03) accounted for a further 7%. These findings indicate that a young age and membership to a horse club or society are associated with favourable control beliefs regarding hoof care behaviour.

#### 3.3.3. Horse Owner Background Factors and Horse Owner Beliefs towards Dental Care Behaviour

The relationships between background factors and beliefs towards dental care behaviour are presented in Table 11. No background factors measured in this study were predictive of behavioural and normative beliefs concerning dental care behaviour. However, *Age* (β = 0.27, *p* = 0.04) was predictive of control beliefs concerning dental care behaviour and accounted for 6% of the variance in the belief. This finding suggests that favourable control beliefs concerning dental care behaviour is associated with an older age.

## 4. Discussion

This paper began to examine the proposed human–horse relationship (Figure 1) by exploring the relationships between horse owner background factors and horse owner beliefs about horse husbandry and management behaviour.

### 4.1. Demographics of the Sample

Comparisons between the background factors (demographic factors, knowledge and experience) of horse owners in the random telephone survey sample (*n* = 206; [25]) and those horse owners in the on-site inspection sample (*n* = 57) indicate largely comparable profiles, with only a number of minor differences identified. Horse owners who completed the on-site inspection appeared more likely to be urban-based, of a younger average age, be members of a horse club or society, have less horse ownership experience (in terms of years), and own fewer horses than those in the larger survey sample. A willingness for further participation in the study, i.e., complete the on-site inspection, may in part be associated with an increased level of commitment to horse ownership, as indicated by the high level of horse club and society membership (over 70%). Furthermore, participants’ age and region of primary residence have both previously been associated with horse club and society membership [3,4,5,25,29], and the high incidence of membership in the on-site inspection sample may explain the observed differences in the age and region of primary residence variables between samples. Thus, while the background factors of the two samples are comparable regarding most variables, the participants who completed the on-site inspection may hold a greater level of commitment to horse ownership.

### 4.2. Relationships between Horse Owner Attributes: Horse Owner Background Factors and Horse Owner Belief Variables

Horse owner attributes including background factors and attitudes (beliefs) towards behaviour are reportedly associated with horse welfare outcomes [3,4,14,15,16]. However, it is unlikely that these attributes share a direct relationship with horse welfare, and this is considered in the second paper investigating the proposed human–horse relationship model (Figure 1). The review by Hemsworth et al. [5] suggests that horse owner attributes are likely to form part of a sequential relationship as hypothesised in Figure 1.

In the present study, we found some relationships between horse owner background factors and attitudes (beliefs) towards horse husbandry and management behaviour. The observed pattern of relationships between these horse owner attribute variables are consistent with the TPB [17] model. We theorised that horse owner salient beliefs (behavioural, normative, and control beliefs), which are assumed to underlie horse owner attitudes towards behaviour, would be associated with a range of background factors. However, only a limited number of relationships were found between horse owner background factors, including demographic factors, knowledge and experience, and beliefs towards horse husbandry and management behaviour.

These findings are also consistent with those reported by Coleman et al. [24] and Waiblinger et al. [30] in the context of livestock, whereby the antecedents of stockperson attitudes to livestock management and handling included demographic factors, knowledge, general attitudes and personality traits. In the current study, attitude subscales and individual belief variables generally correlated significantly with the background factors that were primarily related to knowledge and experience rather than demographic factors. Considering human beliefs are formed from the information an individual possesses about themselves and the world around them [31], the observed relationships between knowledge- and experience-based background factors rather than demographic-based background factors and the belief variables is to be expected. The knowledge- and experience-based background factors, including registered horse ownership, horse club and society membership, and riding instruction, significantly correlated with attitude subscales which concerned beliefs towards behaviour (behavioural belief) and perceived behavioural control about the behaviour (control belief). There was no significant association between any of the background factors and normative belief subscales, suggesting the factors examined may not influence the social pressure horse owners experience regarding horse husbandry and management behaviour.

The attitude subscales (behavioural and control beliefs) associated with background factors were those relating to the performance of horse husbandry and management practices and the resources required for appropriate horse management. The observed positive relationships between the behavioural belief subscales and knowledge- and experience-based background factors indicate that the registration of horse ownership, horse club or society membership, and riding instruction are associated with favourable and realistic attitudes towards appropriate horse husbandry and management practices. Furthermore, the positive relationships observed between the control belief subscales and the knowledge- and experience-based background factors imply that the registration of horse ownership, horse club or society membership, and riding instruction are associated with a greater level of perceived behavioural control with regard to the performance of appropriate horse husbandry and management.

The positive relationships between background factors associated with knowledge and experience and these favourable behavioural and control beliefs may be explained by the opportunity such factors afford the horse owner to both improve their knowledge and reduce their ignorance of appropriate horse husbandry and management practices. Registering horse ownership, horse club and society membership, and obtaining frequent riding instruction are all factors which provide horse owners with the opportunity to access information and support and to interact with fellow horse owners and industry personnel. These activities are likely to offer the horse owner the opportunity to improve their knowledge of appropriate horse husbandry and management practices [3,4].

The primary cause of welfare concerns in recreational horses is believed to be mismanagement by the horse owner, due to ignorance rather than intentional abuse [1,2,3,4,5,6]. As such, it is unsurprising that knowledge- and experience-based background factors rather than demographic-based background factors were associated with horse owner beliefs concerning the appropriate performance of horse husbandry and management behaviour. Importantly, background factors related to knowledge and experience are generally under human control and are therefore able to be modified by the horse owner, while demographic-based background factors lack human control and are consequently difficult, if not impossible, for the horse owner to modify. For example, horse owners are largely able to choose whether they become a member of a horse club or society but are unable to change their gender or age. This finding is important as it demonstrates the potential to improve horse owner beliefs towards behaviour by encouraging the registration of horse ownership, horse club and society membership, and riding instruction in horse owners.

These findings support the first component of the hypothesised sequential relationships between horse owner attributes portrayed in Figure 1. The observed relationships indicate that background factors related to knowledge and experience are associated with both attitudes towards appropriate horse husbandry and management behaviour and perceived behavioural control concerning the appropriate performance of these behaviours. The directions of the observed relationships were predominantly as expected, and imply that background factors which improve knowledge and experience are associated with favourable beliefs about husbandry and management behaviour. These findings are in accordance with the TPB [17] and HAR research from the livestock industries [24,30], and indicate the potential to predict a horse owner’s husbandry and management behaviour from their beliefs towards the behaviours in question.

### 4.3. Predicting Horse Owner Beliefs about Husbandry and Management Behaviour from Horse Owner Background Factors

The observed pattern of relationships between background factors and attitudes (beliefs) towards horse husbandry behaviour are consistent with the TPB [17] model. Consequently, the antecedents of beliefs towards the performance of key husbandry practices were investigated, using *Parasite control behaviour*, *Hoof care behaviour*, and *Dental care behaviour* as target behaviours.

*Age, Riding instruction frequency*, and *Region type* contributed to the variation in horse owner beliefs about parasite control behaviour. A younger age and frequent riding instruction are predictive of favourable attitudes towards parasite control behaviour in horse owners. While an urban region of primary residence and a younger age are predictive of positive subjective norms and favourable perceived behavioural control regarding parasite control.

*Region type*, *Age*, and *Horse club and society membership* contributed to the variation observed in behavioural, normative, and control beliefs concerning hoof care behaviour. A younger age and an urban primary residence are predictive of favourable attitudes towards hoof care. A younger age is also predictive of positive subjective norms regarding hoof care behaviour in horse owners. While a younger age and membership to a horse club or society are predictive of favourable perceived behavioural control regarding hoof care.

*Age* was the only background factor predictive of beliefs concerning dental care behaviour. A younger age is predictive of a positive perceived behavioural control concerning dental care behaviour in horse owners.

There are several possible explanations for the predictive nature of *Age*, whereby younger horse owners appear more likely to have favourable beliefs about the regular performance of both parasite control and hoof care behaviours. First, when compared to younger horse owners, older horse owners may have greater interests or commitments outside of horse ownership. This may result in the need to place less importance on the management of their horses and thus the performance of these two husbandry behaviours. Second, it is possible that the horse ownership of older individuals may be a legacy of their adult children’s childhood ownership. That is, older horse owners may be left to care for their children’s horses after they have lost interest or left home. Results from the random telephone survey [25] suggest that an increase in horse owner’s age corresponds to an increase in horse ownership history and experience. Due to their greater past experience, older horse owners may believe they are more able to independently determine the husbandry and management needs of their horses, and therefore may not follow the recommended guidelines for parasite control and hoof care as closely as those younger horse owners. Consequently, older horse owners may place less importance on the performance of regular husbandry behaviour, may experience less normative pressure, and report a greater inability to perform frequent husbandry behaviour than younger horse owners. In addition, horse owners of a younger age are more likely than older horse owners to be members of horse clubs and societies which is likely to increase their opportunity to interact with other horse owners and industry personnel [25].

These types of interactions associated with *Horse club and society membership* have been shown to have positive effects on horse welfare due to the opportunity for horse owners to improve their knowledge via increased access to information and support [3,4]. This regular interaction with other horse owners and industry personnel may also increase the normative pressure experienced by younger horse owners about regular hoof care behaviour. Interestingly, the nature of the relationship between *Age* and control beliefs concerning dental care behaviour is the opposite of that found for the other two husbandry behaviours. Despite the factors which appear to encourage appropriate husbandry behaviour in younger horse owners, in this case, a young age appears to be predictive of less favourable perceived behavioural control regarding the performance of routine dental care. This may be due to the costs associated with providing dental care to horses. The provision of dental care requires a dentist or veterinarian to administer the treatment, and therefore the associated costs are substantially greater that those involved with regular parasite control or hoof trimming and shoeing. A younger age may be associated with a lower financial status or discretion in expenditure, and as a result limit a horse owner’s ability to provide routine dental care.

The opportunity for interaction between horse owners and industry personnel may also explain the predictive nature of *Riding instruction frequency*. As discussed, the interaction with industry personnel gained through frequent riding instruction may provide horse owners with further opportunity to improve their knowledge of horse husbandry and management, and as such, increase the importance they place on the performance of frequent parasite control behaviour. Furthermore, the opportunity for information and knowledge gain found to be associated with a younger age and membership to a horse club or society could potentially instill confidence in horse owners regarding their ability to appropriately manage parasite control and hoof care in their horses and therefore, increase their perceived behavioural control regarding both husbandry behaviours.

Urban horse owners are more likely than regional horse owners to house their horses at a location separate from their primary residence [25]. Increased separation between the housing location of the horse and the horse owner’s residence may explain the relationship found between *Region type* and beliefs about parasite control and hoof care behaviour. Urban horse owners appear to possess more favorable beliefs concerning the regular performance of the two husbandry behaviours when compared with horse owners residing in regional Victoria. It is possible that urban horse owners may be adopting a more pro-active approach to horse management and the performance of husbandry behaviours than regional horse owners, due to the constraints associated with their type of horse ownership. The ability to house their horses near their primary residence is likely to allow regional horse owners unrestricted access to their horses and a prompt response to a health or welfare problem. Alternatively, the increased separation often associated with urban ownership may restrict a horse owner’s access to their horses and potentially delay their response to these types of problems. As a result, urban horse owners may be more willing to perform frequent husbandry and management practices to prevent or limit the occurrence of potential problems, while regional horse owners may be more prepared to respond to problems if and when they arise. Furthermore, compared with those residing in regional Victoria, urban horse owners are more likely to be of a younger age, have less horse ownership experience, be a member of a horse club or society, and receive frequent riding instruction [25]. The association between these types of factors and the opportunity for knowledge gain has been discussed. An urban horse owner’s increased level of interaction with other horse owners may explain the significant normative pressure they experience regarding parasite control behaviour. In addition, the increased access to information and/or support and the opportunity to improve their knowledge may account for urban horse owners’ increased perception of their volitional control over parasite control behaviour. Additionally, if the urban horse owner is housing their horse at an agistment property, parasite control is likely to be enforced.

Behavioural, normative and control beliefs about hoof care behaviour were predicted to some degree by *Age*, *Region type* and *Horse club and society membership*. However, the limited degree of variation accounted for by these variables suggests that factors not investigated during the current study may be functioning as antecedents to horse owner beliefs about hoof care behaviour. Furthermore, the lack of association between the background factors investigated and beliefs concerning dental care behaviour also suggests that other factors need to be examined. Additional background factors related to knowledge and experience could be examined as potential antecedents of horse owner beliefs as Ajzen [17] reports that information and knowledge are key determinants of an individual’s salient beliefs about behaviour. In addition, the speculative nature of the role of knowledge, knowledge improvement and experience assigned to the observed relationships further supports the need for continued investigation. Further research examining potential antecedents to horse owner beliefs about behaviour and the relationship between knowledge and experience and demographic factors such as horse owner age and region of primary residence is required.

In summary, horse owner behavioural, normative and control beliefs concerning parasite control, hoof care and dental care all appear to be predicted to some degree by knowledge- and experience-based background factors or demographic-based background factors associated with knowledge gain. That is, an aspect common to each of the predictive background factors appears to be the opportunity a horse owner is afforded to interact with other horse owners and potentially improve their knowledge regarding horse husbandry and management. Given that an individual’s salient beliefs are believed to form from the information they possess about themselves and their environment [31], the relationship between background factors associated with knowledge and experience and horse owner beliefs about husbandry behaviour is to be expected. These findings indicate that improving horse owners’ knowledge and experience may improve their beliefs regarding appropriate horse husbandry and management behaviour and thus, potentially reduce the welfare concerns found in recreational horses. However, the speculative nature of the explanations provided indicates that further research is required to continue examining the antecedents to horse owner attitudes (beliefs) towards husbandry and management behaviour.

There remains a clear need for further experimental research examining the human–horse relationship and the subsequent relationship with horse welfare, in order to both improve the welfare of horses and ensure the horse industry’s future sustainability. One of the key issues facing Australian animal use industries is social licence to operate [32] and a key factor underpinning community approval of animal use is the industry’s ability to demonstrate a clear commitment to prioritising and safeguarding the welfare of its animals. As such, the ongoing sustainability of the horse industry and its use of horses in both a competitive and non-competitive capacity, will depend on its ability to demonstrate its commitment to horse welfare.

The antecedents of horse owner husbandry and management behaviour and the ensuing relationship with horse welfare are investigated in a second paper (Hemsworth et al., submitted to Animals) examining the proposed human–horse relationship model outlined in Figure 1.

## 5. Conclusions

The observed relationships between horse owner background factors and horse owner beliefs about horse husbandry and management behaviour largely imply that the antecedents of horse owner attitudes (beliefs) are background factors related to knowledge and experience. Therefore, a practical recommendation is the implementation of education and training programs aimed at improving horse owner knowledge and experience regarding effective horse husbandry and management practices to promote horse welfare. Furthermore, given the current findings, an educational strategy aimed at improving the horse owner’s appreciation of the welfare implications of their behaviour (i.e., targeting their behavioural and control beliefs) may be a component in this strategy. Although the findings of the current study demonstrate relationships between horse owner attributes, experimental work is required to demonstrate the sequential nature of the human–horse relationship and provide evidence of causal relationships, before potential education and training programs to improve the welfare of horses can be developed and evaluated.

## Figures and Tables

**Table 1:** A description of the sections of the random telephone questionnaire.

Questionnaire Section	Questionnaire Content
Section A	This section ascertained whether respondents were horse owners or non-horse owners which, in turn, determined which sections of the questionnaire were completed. Horse owners completed sections A–G, while non-horse owners completed sections A and G only.
Section B	This section collected demographic information about the participants’ horses.
Section C	This section concerned the horse’s environment and the way in which the participant housed their horses.
Section D	This section examined the different types of interactions that occur between the participant and their horses.
Section E	This section assessed the health of the horses and the equine husbandry and management practices employed by the participant.
Section F	This section investigated participants’ attitudes to horses and different horse management and husbandry practices.
Section G	The final section obtained the participant’s demographic details.

**Table 2:** ‘General attitude statements’ attitude subscale from the attitude questionnaire grouped into a composite score, a high score indicates positive attitude or strong agreement to the statements.

Attitude Component	Cronbach’s Alpha	Questionnaire Item
General attitude statements: The horse owner’s view on horses and horse ownership	0.71	I am responsible for my horses’ welfare.
		Horses are not expensive to keep ^(-ve RC)^.
		Horses make great pets.
		Horses are not scary ^(-ve RC)^.
		Horses are affectionate animals.
		Horses are not dangerous ^(-ve RC)^.
		Horses are intelligent animals.
		Horses are beautiful animals.
		Horses are kind animals.

Note: ^(-ve RC)^ re-coded negative attitude item.

**Table 3:** The behavioural belief attitude subscales from the attitude questionnaire grouped into composite scores, a high score indicates positive attitude or strong agreement to the statements.

Attitude Component	Cronbach’s Alpha	Questionnaire Item
Horse husbandry and management–Health and welfare (BB): The horse owner’s attitudes towards the performance of horse husbandry and management practices concerning horse, diet, health, and welfare	0.80	How important is it to base a horse’s diet on its individual needs?
		How important is it to adjust a horse’s diet according to its conditions?
		How often should you check a horse’s condition?
		How important is it to manage and care for a horse according to the work they are doing?
		How important is it to regularly attend to horses’ teeth?
		How often should a horse’s hooves be attended to?
		How important is it to have a veterinarian inspect a horse showing signs of ill-health?
		How important is it to have a horse annually checked by a veterinarian?
		How important is it for the person responsible for a horse to be able to recognise the signs of ill-health and contact a veterinarian for diagnosis and treatment?
		How important is it to recognise, assess, and respond to lameness in horses?
		How important is it to recognise, assess, and respond to injuries in horses?
		How important is it that horse owners know how to look after a horse?
		How important is it to be aware of the possible risks to horses’ welfare?
Horse husbandry and management–Housing (BB): The horse owner’s attitudes towards the performance of horse husbandry and management practices concerning horse housing	0.83	How important is the weather in determining a horse’s water intake?
		How important is it to consider weather conditions when determining which rugs to use?
		How often should you check a horse’s rugs?
		How often should you check and maintain the paddocks horses are kept in?
		How often should you check and maintain a horses’ paddock fencing?
		How important is it that stables do not restrict a horse’s freedom to move?
		How important is it that stables do not restrict a horse’s freedom to lie down?
		How important is it to provide horses with a form of shelter from the wind?
		How important is it to provide horses with a form of shelter from the sun?
		How important is it to provide horses with a form of shelter from the rain?
		How important is it to provide horses with daily supervision?
		How important is it to provide horses with regular exercise or paddock turnout?
Human–horse relationship (BB): The recreational horse owner’s beliefs about the human–horse relationship	0.70	There is always something new to learn about horses.
		Horses provide companionship.
		Horses take up a lot of your time.
		Horses do require a great deal of care ^(-ve RC)^.
		During times of difficulty horses can provide comfort.
		Losing a horse would be a traumatic experience.

Note: ^(-ve RC)^ re-coded negative attitude item.

**Table 4:** The normative belief attitude subscales from the attitude questionnaire grouped into composite scores, a high score indicates positive attitude or strong agreement to the statements.

Attitude Component	Cronbach’s Alpha	Questionnaire Item
Horse husbandry and management–Diet (NB): The horse owner’s normative beliefs about other horse owners’ attitudes towards the performance of horse husbandry and management practices concerning horse diet	0.82	How important do other horse owners believe it is to base a horse’s diet on its individual needs?
		Other horse owners believe that being overweight can be a serious problem for horses.
		How important do other horse owners believe it is to adjust a horse’s diet according to its conditions?
		How important do other horse owners believe it is for horses to have a constant supply of water?
		How important do other horse owners suggest the weather is in determining a horse’s water intake?
Horse husbandry and management–Health and Welfare (NB): The horse owner’s normative beliefs about other horse owners’ attitudes towards the performance of horse husbandry and management practices concerning horse health and welfare	0.90	How often do other horse owners believe you should check a horse’s condition?
		How important do other horse owners believe it is to manage and care for a horse according to the work they are doing?
		How important do other horse owners believe it is to regularly attend to horses’ teeth?
		How often do other horse owners believe a horse’s hooves should be attended to?
		How important do other horse owners believe it is to have a veterinarian inspect a horse showing signs of ill-health?
		How important do other horse owners believe it is to have a horse annually checked by a veterinarian?
		How important do other horse owners believe it is that the person responsible for a horse to be able to recognise the signs of ill-health and contact a veterinarian for diagnosis and treatment?
		How important do other horse owners believe it is to recognise, assess and respond to lameness in horses?
		How important do other horse owners believe it is to recognise, assess and respond to injuries in horses?
		How important do other horse owners believe it is that horse owners know how to look after a horse?
		How important do other horse owners believe it is to be aware of the possible risks to horses’ welfare?
Horse husbandry and management–Housing (NB): The horse owner’s normative beliefs about other horse owners’ attitudes towards the performance of horse husbandry and management practices concerning horse housing	0.90	How important do other horse owners believe it is to consider weather conditions when determining which rugs to use?
		How often do other horse owners suggest you should check your horse’s rugs?
		Do other horse owners believe it is better for a horse to be too hot or too cold? ^(-ve RC)^
		How often do other horse owners believe you should check and maintain the paddocks horses are kept in?
		How often do other horse owners believe you should check a horse’s paddock fencing?
		How important do other horse owners suggest it is that stables do not restrict a horse’s freedom to move?
		How important do other horse owners suggest it is that stables do not restrict a horse’s freedom to lie down?
		How important do other horse owners believe it is to provide horses with a form of shelter from the wind?
		How important do other horse owners believe it is to provide horses with a form of shelter from the sun?
		How important do other horse owners believe it is to provide horses with a form of shelter from the rain?
		How important do other horse owners believe it is to provide horses with daily supervision?
		How important do other horse owners think it is to provide horses with regular exercise or paddock turnout?
Human–horse relationship (NB): The recreational horse owner’s normative beliefs about other horse owners’ attitudes towards the human–horse relationship	0.83	Other horse owners believe that it is my responsibility to provide a safe environment for my horse.
		How important do other horse owners believe it is to manage and care for a horse according to the work they are doing?
		How important do other horse owners believe it is that a horse to responds appropriately to riding aids?
		How important do other horse owners believe it is to be alert when handling horses?
		Other horse owners believe that I am responsible for my horse’s welfare.
		Other horse owners suggest that there is always something new to learn about horses.

Note: ^(-ve RC)^ re-coded negative attitude item.

**Table 5:** The normative belief attitude subscales from the attitude questionnaire grouped into composite scores, a high score indicates positive attitude or strong agreement to the statements.

Attitude Component	Cronbach’s Alpha	Questionnaire Item
Horse husbandry and management–Diet (CB): The horse owner’s control beliefs about how able they are to perform horse husbandry and management practices concerning horse diet	0.70	To what extent are you able to base your horses’ diet on its individual needs?
		How difficult is it for you to ensure your horse does not become too fat?
		How difficult is it for you to adjust your horse’s diet according to its conditions?
		How difficult is it for you to provide your horse with a constant supply of water?
Horse husbandry and management–Health and welfare (CB): The horse owner’s control beliefs about how able they are to perform horse husbandry and management practices concerning horse health and welfare	0.80	How often are you able to check your horses’ condition?
		To what extent are you able to manage and care for your horse in a manner suitable for the work they are performing?
		How often are you able to treat your horses for worms?
		How difficult is it for you to have your horse’s teeth regularly attended to?
		How often are you able to attend to your horse’s hooves?
		How difficult is it for you to have a veterinarian inspect your unwell horse?
		How difficult is it for you to have a veterinarian annually check your horse?
		How difficult would it be for you to recognise the signs of ill-health in your horse and contact a veterinarian for diagnosis and treatment?
		How difficult is it for you to recognise, access and respond to lameness in your horses?
		How difficult is it for you to recognise, access and respond to injuries in your horses?
		To what extent do you know how to look after a horse?
		To what extent are you aware of the possible risks to horses’ welfare?
Horse husbandry and management–Housing (CB): The horse owner’s control beliefs about how able they are to perform horse husbandry and management practices concerning horse housing	0.81	To what extent can you choose rugs to use according to the weather?
		How often are you actually able to check your horse’s rugs?
		How difficult is it for you to determine the correct temperature for your horse when using rugs?
		To what extent are you able to provide your horse with a safe environment?
		How often can you check and maintain the paddocks your horse is kept in?
		How often are you able to check and maintain your horse’s paddocks fencing?
		How often can you check and maintain the stables your horse is kept in?
		How difficult is it for you to ensure that stables do not restrict your horse’s freedom to move?
		How difficult is it for you to ensure that stables do not restrict your horse’s freedom to lie down?
		How difficult is it for you to provide your horse with shelter from the wind?
		How difficult is it for you to provide your horse with shelter from the sun?
		How difficult is it for you to provide your horse with shelter from the rain?
		How often are you able to check your horses’ condition?
		How difficult is it for you to provide your horse with daily supervision?
		How difficult is it for you to provide your horses with regular exercise or paddock turnout?
		How difficult is it for you to provide your horse with social contact from other horses?
Human–horse relationship (CB) The horse owner’s control beliefs about how able they are to perform behaviours regarding human–horse relationship	0.62	How difficult is it for you to ride your horse?
		To what extent are you able to ensure you have good basic riding skills?
		How difficult is it for you to be responsible for your horse’s welfare?
		How difficult is it for you to access information and assistance from industry personnel to improve the way you handle and care for your horses?
		How difficult is it for you to learn new things about horses?

Note: ^(-ve RC)^ re-coded negative attitude item.

**Table 6:** Horse owner background factors (by percentage %) (*n* = 57).

Type of Background Factor	Background Factor		Horse Owner %
Demographic factors	Participant location	City/Urban	16
		Regional	74
	Region of primary residence	City	2
		Urban	10
		Peri-urban	4
		Semi-rural	33
		Rural	51
	Gender	Female	86
		Male	14
	Age (years)	<18	9
		18–25	4
		26–35	21
		36–45	28
		46–55	19
		56–65	12
		65+	7
	Children	Yes	77
		No	23
	Level of education	Did not complete high school	25
		Secondary education	33
		Tertiary education	33
		Post-tertiary education	5
		TAFE course	4
		Other (undefined)	0
	Field of occupation	Professional	30
		Non-professional	12
		Trades and services	4
		Student	11
		Retired	9
		Unemployed	0
		Domestic	9
		Other (undefined)	26
	Household annual income (before tax)	<$20,000	6
		$20,000–35,000	17
		$36,000–50,000	6
		$51,000–70,000	29
		$71,000–1000,000	21
		>$100,000	21
	Residence type	Apartment/townhouse	0
		Small land block	17
		Large land block	23
		Acreage	60
Knowledge and experience	Horse club or society member	Yes	74
		No	16
	Registered horse owner	Yes	41
		No	59
	Actively improve knowledge	Yes	83
		No	17
	Awareness of the Codes of Practice pertaining to horse welfare in Victoria	Yes	47
		No	53
	Riding instruction	Yes	72
		No	28
	Participation in horse competitions	Yes	54
		No	46

Note: TAFE (Technical and Further Education) refers to Government run vocational education and training. Riding instruction refers to the horse owner reporting that they have received riding instruction. Actively improve knowledge refers to the horse owner reporting that they actively seek to improve their knowledge of horses, horse husbandry and management, and horse welfare.

**Table 7:** Demographic statistics of horses (*n* = 98; H1 *n* = 57, H2 *n* = 41).

Variable		Sample %	H1 %	H2 %
Horse sex	Mare/filly	47	40	50
	Gelding	48	53	45
	Stallion/colt	5	7	5
Horse age (years)	<1	6	7	5
	1–4	9	4	17
	5–10	30	32	27
	11–15	27	30	22
	16–20	12	11	16
	21–25	9	11	5
	26 +	6	5	8
Horse breed	Arabian	6	4	10
	Australian stock horse	3	2	5
	Crossbred	4	5	2
	Pony	8	9	7
	Quarterhorse	2	4	0
	Standard bred	10	12	7
	Thoroughbred	18	16	22
	Thoroughbred (ex-racehorse)	12	12	12
	Thoroughbred (un-raced)	6	4	10
	Warmblood	11	11	12
	Other	20	23	17
Region where horse is housed	Peri-Urban	15	16	14
	Regional/Rural	75	84	86

**Table 8:** Descriptive data regarding the horse management and husbandry practices implemented by horse owners (*n* = 98; H1 *n* = 57, H2 *n* = 41).

Variable		Sample %	H1 %	H2 %
Horses location	Home/Primary residence	70	67	71
	Owners land, away from primary residence	10	9	12
	Agistment property	13	16	10
	Family/friends property	8	7	7
	Full-time paddock housing	80	75	83
Daily interaction between owner and horse	Yes	79	79	79
	No	21	21	21
Does the farrier attend to horse’s hooves	Yes	84	84	83
	No	1	2	0
	No, owner attends to horse’s hooves	15	14	17
How often are horse’s hooves attended to	Never	1	2	0
	Monthly	11	11	12
	Every 6–8 weeks	62	67	57
	Every 3 months	16	12	21
	Every 6 months	4	5	2
	Yearly	5	4	7
How often is horse treated for worms	Never	4	4	5
	Monthly	6	5	7
	Every 6–8 weeks	27	28	26
	Every 3 months	40	40	38
	Every 6 months	20	18	21
	Yearly	4	5	4
Dental technician attends to horse’s teeth	Yes	85	84	86
	No	15	16	14
Owners rating of horses BCS	Very thin	0	0	0
	Thin	1	2	0
	Correct weight	59	61	57
	Overweight	35	33	38
	Very overweight	4	4	5
Supplementary feeding	No supplementary feeding	14	9	21
	Roughage	33	32	36
	Grain/processed feed	2	2	2
	Roughage and Grain/processed feed	50	58	41
Presence of an injury or illness	Yes	29	35	21
	No	71	65	79
Health concerns in last 12 months	Yes	26	35	15
	No	74	65	85
Veterinary attention	Yes	54	58	48
	No	46	42	52
Horse registered	Yes	47	47	46
	No	53	53	53
Horse ridden	Yes	58	67	42

**Table 9:** Pearson product-moment correlations (*p* < 0.05) between horse owner demographic-based background factors and attitude subscales, individual behavioural belief variables (A variables), individual normative belief variables (B variables), and individual control belief variables (C variables) (*n* = 57).

Attitude Variable	Region Type	Age	Gender	Children	Prop Type	Prop Size	Horse #	Animals
Gen A		0.30 *						
BB hw								
BB int								
NB d		−0.36 *						
CB hw								
CB h								
A9			0.30 *					
A18								
A19								
A22								
A28	−0.29 *	−0.48 **						
A30	0.35 **	0.43 **	−0.38 *		0.32 **			
A33				0.33 **		0.27 *		
A34		−0.27 *		0.27 *				
A42								
A43								
A46			−0.36 **				−0.30 *	
B1		−0.33 *						
B3		−0.34 *						
B9								
B28	0.33 **	−0.32 *						
B30	0.37 **			−0.30 *				
B31								−0.41 **
B37								
B41								
B42								
B43								
C18						0.38 **		
C19								−0.28 *
C28	0.33 **	0.52 **						
C29		0.27 *	−0.26 *					
C30		0.33 **	−0.27 *					
C34								
C36								0.27 *
C41								
C42						−0.32 **		
C43								−0.31 *

Note: * *p* < 0.05 ** *p* < 0.01, a blank space indicates *p* > 0.10, df = 55. ^(-ve RC)^ re-coded negative attitude item. Background factors–demographic factors: Region type refers to the region type where the horse owner’s primary residence is located, Age refers to the horse owner’s age, Gender refers to the horse owner’s gender, Children refers to whether the horse owner has children, Prop type refers to property type, Prop size refers to property size, Horse # refers to number of horses, Animals refers to animals other than horses. Attitude items: Gen A refers to General attitude statements, BB hw refers to Horse husbandry and management-health and welfare (Bb), BB int refers to human–horse interaction (Bb), NB d refers to horse husbandry and management–diet (Nb), CB hw refers to horse husbandry and management–health and welfare (Cb), CB h refers to horse husbandry and management–housing (Cb), A9^(-ve RC)^. It is my responsibility to provide a safe environment for my horse, A18. How often should you check a horse’s condition? A19. How important is it to provide horses with daily supervision? A22. How important is it to manage and care for a horse according to the work they are doing? A28^(-ve RC)^. How often should you treat horses for worms? A30. How often should a horse’s hooves be attended to? A33. How important is it for the person responsible for a horse to be able to recognise the signs of ill-health and contact a veterinarian for diagnosis and treatment? A34. How important is it to recognise, assess and respond to lameness in horses? A42. Industry personnel can provide information and assistance that can improve the way we handle and care for horses, A43. There is always something new to learn about horses, A46. Horses are difficult to look after, B1. How important do other horse owners believe it is to base a horse’s diet on its individual needs, B3. How important do other horse owners believe it is to adjust a horse’s diet according to its conditions? B9. Other horse owners believe that it is my responsibility to provide a safe environment for my horse, B28. How often do other horse owners suggest that horses should be treated for worms? B30. How often do other horse owners believe a horse’s hooves should be attended to? B31. How important do other horse owners believe it is to have a veterinarian inspect a horse showing signs of ill-health? B37. How important do other horse owners believe it is to be aware of the possible risks to horses’ welfare? B41. Other horse owners believe that I am responsible for my horse’s welfare, B42. Other horse owners believe that industry personnel can provide information and assistance that can improve the way we handle and care for horses, B43. Other horse owners suggest that there is always something new to learn about horses, C18. How often are you able to check your horse’s condition? C19. How difficult is it for you to provide your horse with daily supervision? C28. How often are you able to treat your horses for worms? C29. How difficult is it for you to have your horse’s teeth regularly attended to? C30. How often are you able to attend to your horse’s hooves? C34. How difficult is it for you to recognise, access and respond to lameness in your horses? C36. To what extent do you know how to look after a horse? C41. How difficult is it for you to be responsible for your horse’s welfare? C42. How difficult is it for you to access information and assistance from industry personnel to improve the way you handle and care for your horses? and C43. How difficult is it for you to learn new things about horses?

**Table 10:** Pearson product-moment correlations (*p* < 0.05) between horse owner knowledge- and experience-based background factors and attitude subscales, individual behavioural belief variables (A variables), individual normative belief variables (B variables), and individual control belief variables (C variables) (*n* = 57).

Attitude Variable	HCS Member	Reg Owner	Own Yrs	Ride Instruct	Ride Int freq	Aware CoP	Improve Knowledge
Gen A	−0.29 *						
BB hw						0.32 *	
BB int				0.33 *			
NB d							
CB hw	0.29 *	0.30 *					
CB h							0.38 **
A9				−0.27 *			
A18						0.33 **	
A19		0.28 *					
A22						0.32 *	
A28					−0.56 **		
A30	0.32 *			0.28 *			
A33							
A34	−0.27 *						
A42						−0.31 *	
A43			0.27 *			0.29 *	
A46	0.31 *						
B1							
B3							
B9				0.30 *			
B28							
B30				0.32 **			
B31							
B37				0.28 *			
B41							0.33 **
B42							0.31 *
B43							0.30 *
C18							
C19							
C28					0.43 **		
C29		0.27 *			0.44 **		
C30		0.36 **					
C34			−0.36 **				
C36		0.30 *					
C41				0.30 *			
C42			−0.26 *				
C43							

Note: * *p* < 0.05 ** *p* < 0.01, a blank space indicates *p* > 0.10, df = 55. ^(-ve RC)^ re-coded negative attitude item. Background factors–knowledge and experience: HCS Member is an abbreviation of horse club and society member, Reg owner refers to registered horse owner, Own yrs refers to horse ownership years, Ride instruct refers to riding instruction, Ride int freq refers to the frequency of riding instruction, Aware CoP refers to awareness of the Code of Practice pertaining to the welfare of recreational horses and Improve knowledge refers to the active improvement of knowledge. Attitude items: Gen A refers to General attitude statements (positive), BB hw refers to horse husbandry and management–health and welfare (Bb), BB int refers to human–horse interaction (Bb), NB d refers to horse husbandry and management–diet (Nb), CB hw refers to horse husbandry and management–health and welfare (Cb), CB h refers to horse husbandry and management–housing (Cb), A9^(-ve RC)^. It is my responsibility to provide a safe environment for my horse, A18. How often should you check a horse’s condition? A19. How important is it to provide horses with daily supervision? A22. How important is it to manage and care for a horse according to the work they are doing? A28^(-ve RC)^. How often should you treat horses for worms? A30. How often should a horse’s hooves be attended to? A33. How important is it for the person responsible for a horse to be able to recognise the signs of ill-health and contact a veterinarian for diagnosis and treatment? A34. How important is it to recognise, assess and respond to lameness in horses? A42. Industry personnel can provide information and assistance that can improve the way we handle and care for horses, A43. There is always something new to learn about horses, A46. Horses are difficult to look after, B1. How important do other horse owners believe it is to base a horse’s diet on its individual needs, B3. How important do other horse owners believe it is to adjust a horse’s diet according to its conditions? B9. Other horse owners believe that it is my responsibility to provide a safe environment for my horse, B28. How often do other horse owners suggest that horses should be treated for worms? B30. How often do other horse owners believe a horse’s hooves should be attended to? B31. How important do other horse owners believe it is to have a veterinarian inspect a horse showing signs of ill-health? B37. How important do other horse owners believe it is to be aware of the possible risks to horses’ welfare? B41. Other horse owners believe that I am responsible for my horse’s welfare, B42. Other horse owners believe that industry personnel can provide information and assistance that can improve the way we handle and care for horses, B43. Other horse owners suggest that there is always something new to learn about horses, C18. How often are you able to check your horse’s condition? C19. How difficult is it for you to provide your horse with daily supervision? C28. How often are you able to treat your horses for worms? C29. How difficult is it for you to have your horse’s teeth regularly attended to? C30. How often are you able to attend to your horse’s hooves? C34. How difficult is it for you to recognise, access and respond to lameness in your horses? C36. To what extent do you know how to look after a horse? C41. How difficult is it for you to be responsible for your horse’s welfare? C42. How difficult is it for you to access information and assistance from industry personnel to improve the way you handle and care for your horses? and C43. How difficult is it for you to learn new things about horses?

**Table 11:** Stepwise linear regression analyses for horse owner beliefs about the performance of three key husbandry behaviours and horse owner background factors (*n* = 57).

Horse Owner Belief	Background Factor	Zero-Order Correlation	β Coefficient	Standard Error	Adjusted R^2^	*p*-Value
*Parasite control*						
Behavioural belief A28	Age	−0.48	−0.48	0.50	0.20	0.01
	Ride int freq	−0.56	−0.43	0.47	0.11	0.02
Normative belief B28	Region type	0.33	0.33	0.55	0.09	0.01
	Age	0.32	0.26	0.53	0.05	0.04
Control belief C28	Age	0.59	0.59	0.47	0.32	0.00
	Region type	0.30	0.33	0.43	0.09	0.02
*Hoof care*						
Behavioural belief A30	Region type	0.35	0.35	0.54	0.11	0.00
	Age	0.43	0.37	0.50	0.12	0.00
Normative belief B30	Age	0.42	0.42	0.74	0.15	0.01
Control belief C30	Age	0.33	0.33	0.59	0.09	0.01
	HCS Member	0.36	0.29	0.56	0.07	0.03
*Dental care*						
Behavioural belief A29						
Normative belief B29						
Control C29	Age	0.27	0.27	0.84	0.06	0.04

Note: ^(-ve RC)^ re-coded negative attitude item, Beliefs concerning parasite control: A28^(-ve RC)^. How often should you treat horses for worms? B28. How often do other horse owners believe you should treat horses for worms? C28. How often are you able to treat your horses for worms? Beliefs regarding hoof care behaviour: A30. How often should a horse’s hooves be attended to? B28. How often do other horse owners believe a horse’s hooves should be attended to? C28. How often are you able to attend to your horse’s hooves? Beliefs regarding dental care behaviour: A29. How important is it to regularly attend to horses’ teeth? B29. How important do other horse owners believe it is to attend to horses’ teeth? C29. How difficult is it for you to have your horse’s teeth regularly attended to? Background factors–demographic factors: Age refers to the horse owner’s age, Children refers to whether the horse owner has children, Gender refers to the horse owner’s gender, Region type refers to the region type where the horse owner’s primary residence is located. Background factors–knowledge and experience: HCS Member is an abbreviation of horse club and society member, Ride int freq refers to the frequency of riding instruction.

## Data Availability

The data presented in this study are available on request from the corresponding author.
